# Pasture Heterogeneity Improves Donkey Welfare: Effects of Structural Variation, Species Diversity, and Sward Height on Herd Emotional States

**DOI:** 10.3390/ani15233421

**Published:** 2025-11-27

**Authors:** Jessie Fitts, Laura M. Kubasiewicz, Stuart L. Norris, Sarah Worth, Tamlin Watson, Ruth L. Angell, Mark D. Steer, Paul Lintott

**Affiliations:** 1 The Centre for Sustainable Agri-Food and Environment, University of the West of England, Bristol BS16 1QY, UK; mark.steer@uwe.ac.uk (M.D.S.);; 2 The Donkey Sanctuary, Sidmouth EX10 0NU, UK; laura.kubasiewicz@thedonkeysanctuary.org.uk (L.M.K.);

**Keywords:** donkey, welfare, Qualitative Behaviour Assessment, equid welfare, habitat heterogeneity

## Abstract

Grazing environment plays a key role in the welfare of domestic herbivores. In the UK, donkeys are typically kept on species-poor, agriculturally improved grasslands that offer limited dietary and behavioural opportunities. Obesity, driven by low exercise levels and unsuitable diet, remains a primary welfare concern in this species. This study examined whether field habitat heterogeneity, measured as structural variation, botanical diversity, and sward height, affects donkey welfare. Over a six-month grazing season, three donkey herds rotationally grazed 10 fields that differed in their level of habitat heterogeneity. Animal herd welfare was assessed using Qualitative Behaviour Assessment (QBA) of 194 videos, each scored independently by three trained enumerators. Habitat heterogeneity had a highly significant influence on donkey herd emotional states. Fields with greater structural variation were associated with more energetic behavioural expressions, while taller swards were linked to calmer, more relaxed states. Taller grass may have reduced competition for resources, leading to a more relaxed herd, while structural variation created environmental differences that encouraged more energetic expressions. These findings demonstrate that increasing field habitat heterogeneity can promote positive welfare states in donkeys, highlighting the importance of integrating environmental or semi-natural habitat features into grazing management practices.

## Simple Summary

The grazing environment plays an important role in the behaviour and welfare of donkeys. In the UK, donkeys are often kept on homogeneous pastures with little dietary choice or opportunity to browse. Unsuitable diets and habitats can contribute to low activity levels and obesity, a primary welfare concern for donkeys. In this study, we explored whether access to more varied pasture environments affected donkey herd wellbeing. Donkey herds grazed a range of fields that differed in grass height, plant diversity, and access of shrubs and trees. We found that herds in more diverse fields showed more positive emotional states. Taller grass was linked to calmer and relaxed donkey herds, while fields with more shrubs and trees encouraged energetic expressions. Our findings suggest that creating more diverse grazing environments can benefit donkey welfare and should be considered in management.

## 1. Introduction

Animal welfare is closely linked to the animal’s physical environment, with more natural and complex habitats promoting species-typical behaviours and better welfare outcomes [1,2]. Studies on zoo enclosures [3,4], farms [5], extensive management settings [6,7], and enrichment trials [8,9,10] consistently show that increased environmental complexity enhances behavioural and overall wellbeing. Complex environments can provide animals with greater freedom of choice to display behaviours and select diet, leading to improvements in animal welfare indicators such as increased natural behaviour expression [11]. For example, horses allowed access to more natural environments will demonstrate foraging and social behaviours similar to their wild counterparts [12]. Yet, many domestic animals are kept in environments that restrict their quality of life and lead to poorer welfare [13]. Modifying domestic environments to suit an animal’s evolved ecology is therefore critical to improving animal welfare [13,14]. However, despite the broad understanding of the links between environment and welfare, the specific influence of habitat heterogeneity, particularly in donkeys (*Equus asinus*), remains largely unexplored.

An estimated 52.9 million donkeys inhabit a wide range of environmental conditions globally, with populations of free-roaming and working donkeys found across the globe [15,16]. In the UK, donkeys are primarily kept as companion animals, where their activity levels and environmental conditions often result in distinct welfare issues. One in three donkeys in the UK are overweight as a result, at least in part, of their environment, unsuitable diet, and low activity levels [17,18]. Without careful management, obesity can lead to further health problems such as hyperlipemia and laminitis [19,20]. Upon relinquishment to sanctuary care in the UK, 2013–2015 intake data identified 34.9% of donkeys as overweight or obese, and the behaviour and health of companion donkeys were among the top reasons for relinquishment [21].

Donkeys evolved from the African wild ass (*Equus africanus*) in arid and semi-arid habitats with nutrient-poor forage, making them efficient browsers and grazers of fibrous vegetation [22,23,24]. As hind-gut fermenters, donkeys can continuously digest plant material and spend 14–18 h a day browsing and grazing [25]. Evident in free-roaming conditions, donkeys will consume shrubs and woody species, and these materials can account for up to 30% of their diet [26,27]. Browsing is a key adaptive behaviour for donkeys, so a lack of accessible browse could negatively impact their behaviour and lead to compromised welfare.

In the UK, donkeys are often grazed on lush or degraded agriculturally improved pastures with low browse opportunities and limited botanical diversity [25]. Many UK donkey owners apply horse feeding guidelines, even though donkeys have lower dry matter intake (DMI) and longer gut retention times [28,29]. High intake of energy-rich forage with inappropriate grazing has resulted in an ‘obesity crisis’ for UK equids, including donkeys, and access to pasture is recognised as a contributing risk factor [18,21,30,31]. Whilst environmental modifications can provide opportunities for donkeys to express natural behaviours such as foraging, selection, and social interaction [17,25], few studies offer species-specific practical guidance on how the provision of browse and access to a complex environment might improve donkey welfare.

Grasslands cover 40% of land in the UK and have the potential to be one of the most species-rich habitats in Europe [32]. However, intensive agricultural practices have resulted in homogeneous, species-poor landscapes [32,33]. Grazing at a low-to-moderate stocking density can increase plant diversity by creating patches through selective foraging, trampling, and nutrient distribution [14,34]. Increasing grassland heterogeneity enhances vegetation structure, species richness, and overall plant diversity, providing grazers such as sheep and cattle with greater foraging choice and opportunities to express natural behaviour [35,36]. Both the forage quality and quantity influence grazer behaviour, with preferences often driven by relative availability [32,37]. Appropriate stocking densities are therefore recommended to ensure animals can access sufficient resources and support their welfare [13,32]. Whilst recent research has demonstrated that access to grazing improves equine welfare [1,38], these studies have primarily focused on horses [7].

A wide range of welfare measurements have been used to assess animals on pasture [39], for example, monitoring the behaviour and health of leisure horses [1,32]. For donkeys, multiple welfare assessments have been developed, with some more relevant to the animal’s role as either a companion or working animal [40]. Animal welfare indicators (AWINs) aim to promote the welfare of animal species by developing practical welfare assessments. Qualitative Behaviour Assessment (QBA) is part of the second level of the AWIN protocol and is commonly used to monitor welfare of donkeys at The Donkey Sanctuary, UK. QBA uses a predeveloped list of terms created by species experts to describe the affective state of the donkey or donkey herd. The method has proven a suitable tool to assess the welfare of donkeys on farms, along with validation across multiple animal species [41].

Although free-roaming donkeys will consume a variety of vegetation and use different vegetation mosaics [42], there is currently no research into the grazing behaviours of managed donkeys and how grazing environments impact donkey welfare. To address this gap, we investigated the relationship between habitat heterogeneity and donkey welfare. We hypothesised that increased field habitat heterogeneity would be associated with more positive emotional states. Specifically, we aimed to achieve the following:

1. Assess how overall field-level habitat heterogeneity influences herd emotional state;
2. Examine how individual components of habitat heterogeneity are associated with variation in emotional states.

## 2. Materials and Methods

### 2.1. Study Area

**Figure 1:**
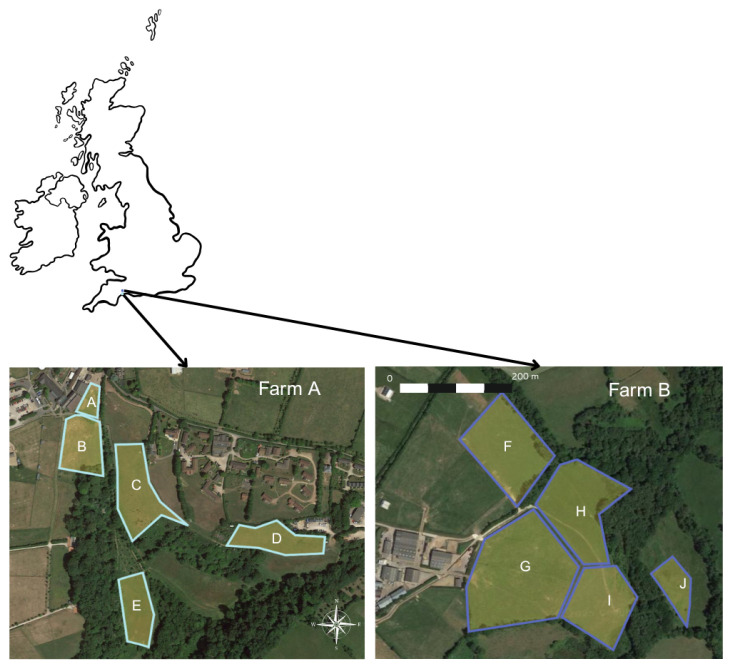
Fields (A–J) used in study across two Donkey Sanctuary sites in Devon, UK, based on rotational grazing schedule. Image source: Google Earth © Google; imagery © Airbus, 18 June 2025.

This study was carried out from April to September 2024 across two farms (farm A and farm B) in Devon, United Kingdom (50.697° N, 3.191° W and 50.775° N, 3.178° W, respectively), both part of The Donkey Sanctuary, a UK-based Non-Government Organisation (NGO). Local weather conditions during the study period from the nearest national meteorological station (10 miles from study site) showed mean monthly temperatures ranging from 9.8 °C to 17.3 °C and monthly rainfall from 11.4 mm to 76.4 mm [43]. Three herds (one at farm A and two at farm B) rotationally grazed a subset of 10 fields total throughout the season (Figure 1). Herds only had access to fields part of their respective farms, and fields were never grazed by more than one herd simultaneously or on the same day.

Most fields had been grazed by donkeys in previous years, except for one field (Figure 1; Field J), which had only been sheep-grazed for the 10 years prior to the study.

When not used by donkeys, fields were rested or lightly grazed by sheep during the winter. Allocation of donkey herds to fields followed an existing grazing schedule determined by farm management, based on weather conditions and the standard practice of rotating fields when sward height reached 5 cm. Grazing duration in each allocated field ranged from one week to one month, with some herds returning to previously grazed fields later in the season. Each field was grazed by a single herd, with the exception of field H (Figure 1), which was grazed by two herds at separate times (Table 1).

### 2.2. Animals and Management

A total of 215 donkeys with a mean age of 14 ± 3.8 within three herds were included in this study (Table 2). Farm A was open to the public, while farm B was limited to Donkey Sanctuary staff. All donkeys had 24 h access to shelters and ad lib straw in troughs within shelters. Prior to the study period, donkeys were housed in shelters during winter months. The study herds were selected due to their rotational grazing schedule and the absence of supplementary feed or additional medical needs. Donkey herds either had daily 24 h access to grazing (herd 1) or daytime access (herd 2 and herd 3). The donkeys in all herds followed The Donkey Sanctuary routine farrier, dental, weighing, vaccination, and worming schedule.

### 2.3. Habitat Assessment

#### 2.3.1. Structural Variation

Structural variation, the amount of variability in the physical structure of a whole habitat, was assessed in all fields. Systematic field-walking surveys were conducted to identify key structural features including the presence of scrub, hedge (within field or alongside perimeter), trees (within or along perimeter), elevation change (e.g., hills), and fallen logs. Browse accessibility was assessed along field fences and included in the structural variation score based on whether the vegetation was reachable by donkeys through and above fences. Each field was assigned a structural variation score on a scale of 1–4, reflecting the amount and range of different habitat features per field, with 1 being low and 4 being high. Full scoring criteria and photos are provided in the Supplementary Materials in Table S1 and Figures S1–S4.

#### 2.3.2. Species Abundance, Species Richness, and Sward Height

Following an across-farm survey of all fields prior to commencing field work to establish a base level habitat heterogeneity, a total of 38 botanical surveys were conducted over the summer grazing period. To account for seasonal variation in vegetation growth, grazed fields were surveyed at intervals of no more than two weeks. Surveys aligned with donkey use in each field and were only conducted if grazing occurred during the welfare measurement time frame.

During each survey, 10 1m^2^ quadrats were placed randomly along a W-shaped transect within the field. For each quadrat, the relative abundance of each species was identified estimated using the percentage cover of each species, species richness was estimated by the number of species present, and average sward height was measured.

### 2.4. Donkey Welfare Assessment

Donkey herd-level welfare was assessed using Qualitative Behaviour Assessment (QBA) based on methods developed by Minero et al. [41]. A total of 196 three-minute videos were collected across all treatments and herds. Recordings were conducted weekly for the duration of the study period, other than on occasions when donkey herds were inside shelters. Criteria for conducting video observation consisted of identifying one or more individuals in the herd grazing in field; however, behaviours and grazing duration prior to recording were unknown as donkeys had free access to shelters. Weekly recording sessions were adjusted to maximise the best weather to increase the probability of donkeys choosing to be outdoors rather than inside shelters.

All videos were recorded using either a tablet or iPhone 14 device (Apple Inc., Cupertino, CA, USA) between 9 am and 5 pm when farms were open to staff. Each recording captured as many individuals as possible within the herd to provide a representative assessment of overall herd behaviour. All recording was conducted in a fixed position, moving only when necessary to avoid interactions with donkeys and to follow donkeys when they moved out of view. Before filming began, the observer remained stationary to allow the donkeys to acclimate to their presence and ensure no interaction occurred. All donkey herds had regular human contact prior to the study; therefore, the presence of the observer was not considered novel. Based on established protocols for QBA in herd environments, between six and eight videos were recorded per herd per study day to ensure accurate representation of behavioural states [5,44,45]. If only part of the herd was grazing, fewer videos were recorded to reflect the proportion of herd present following Rousing and Welmelsfelder [5]. The Donkey Sanctuary utilise QBA for many on-farm welfare assessments, as it supports the evaluation of the behavioural expression of the whole animal, especially as QBA is the only known indicator to feasibly measure emotional state [41]. Some studies have highlighted issues with this method, noting potential problems with observer bias and subjectivity. These problems were mitigated by enumerator training, measuring reliability, and blind scoring [46,47]. QBA descriptors used for scoring were derived by Minero et al. [41], with a full list of descriptors available in the Supplementary Materials in Table S2.

### 2.5. Data Analysis

The Shannon diversity index, the estimate of species diversity using number of species and their relative abundance, was calculated for each quadrat. The means of the Shannon diversity indexes and sward height measurements per quadrat were used to generate diversity information for each field survey [48,49,50].

All three diversity indicators (structural variation, average Shannon’s diversity index score across all quadrats, and average sward height across all quadrats) were combined to obtain an index of total habitat heterogeneity for each field. To ensure comparability and equal contribution of each component, all three diversity indicators were standardised by converting them into Z-scores prior to calculating the composite habitat heterogeneity index. Based on the distribution of habitat heterogeneity scores, a threshold method using natural breaks was used to categorise low, mid, and high habitat heterogeneity for analysis. Although individual components of habitat heterogeneity do not fit a completely linear pattern within the final groupings, their combined values are the best fit for each category.

QBA videos were assessed by three trained enumerators, where enumerators underwent a three-stage training consisting of QBA theory, application familiarity, and data processing. Training included scoring 10 example videos for comparison with other trained enumerators to assess inter-observer reliability. Once consistent reliability was obtained, enumerators independently scored the full dataset of pre-recorded videos. Inter-observer agreement was assessed using Kendall’s Coefficient of Concordance (W) with a threshold of W < 0.60 set for agreement levels [41].

All assessments were completed on a Samsung Galaxy Tab A (T580) (Samsung Electronics, Suwon, Public of Korea) with the Android application Open Data Kit (ODK) collect v2024 1.3 (Open Data Kit, University of Washington, Seattle, WA, USA) [51], with each emotional descriptor scored from 0–125. Observers were blind to the treatment group and scored videos without access to their own scores or those of other assessors so they could not change scores. Videos were presented in a randomised order and scored in increments to reduce potential order and fatigue effects. Although enumerators were familiar with some area fields, no videos were scored live or included any information about field diversity measures.

#### 2.5.1. Habitat Heterogeneity and Donkey Emotional State

To understand the relationship between habitat heterogeneity and donkey emotional state, we conducted a principal component analysis (PCA) to identify the main dimensions of variance in emotional state, and the first two components PC1 (mood) and PC2 (energy) were used for further investigation. To assess differences in emotional expression across habitat heterogeneity groups, a multivariate analysis of variance was conducted using PERMANOVA (adonis2), which calculates a pseudo-F statistic based on allocating sums of squares from a Euclidean distance matrix. Significance was determined via permutation testing with Bonferroni’s adjusted *p*-values used for pairwise comparisons. QBA scores were log-transformed prior to analysis to meet assumptions of Euclidean distance. Effects of habitat heterogeneity groups on descriptors were further explored through permutation testing with SIMPER.

#### 2.5.2. Components of Habitat Heterogeneity

Two linear models were used to examine the impact of individual diversity measures (structural variation, Shannon’s diversity index, and sward height) on PC1 (mood) and PC2 (energy) scores derived from the PCA. Prior to model fitting, Pearson’s correlation analysis was conducted between diversity variables to assess multicollinearity. No strong correlations were found between predictors in the models used (all r < 0.4). Both models assumed a normal distribution of residuals. Model assumptions were assessed through visual representation of residuals to test for normality and homoscedasticity using diagnostic plots including histograms, residual vs fitted, and Q-Q plots. PC1 residuals violated normality assumptions, so a log transformation was applied to the PC1 scores. To enable comparison of effect sizes across habitat heterogeneity components and between mood and energy models, both predictors and response variables were z-transformed. Scaled coefficients represent the change in mood and energy (in standard deviations) for a one-standard-deviation increase in the habitat heterogeneity component.

To test whether underlying behavioural differences among herds or farms could influence QBA scores, post hoc PERMANOVA (adonis2) was conducted on a Euclidean distance matrix of log-transformed QBA scores, with herd and farm as factors. Significance was assessed via permutation testing, and pairwise comparisons were performed with Bonferroni-adjusted *p*-values.

All analyses were completed using the R (R Core Team, Vienna, Austria, 2022) and RStudio (v.4.3.3) packages vegan, argricolae, ggplot2, and tidyverse, with a significance level of *p* < 0.05 applied throughout [52].

## 3. Results

### 3.1. Scoring and Inter-Observer Agreement

Observers showed high agreement in their scoring (Kendall’s W averaging 0.85; SD = 0.07), indicating consistent interobserver reliability. The two videos that had low agreement (W < 0.60) were removed from the analysis based on threshold criteria [41], retaining 194 videos for further analysis.

### 3.2. Habitat Heterogeneity Categorisation

**Figure 2:**
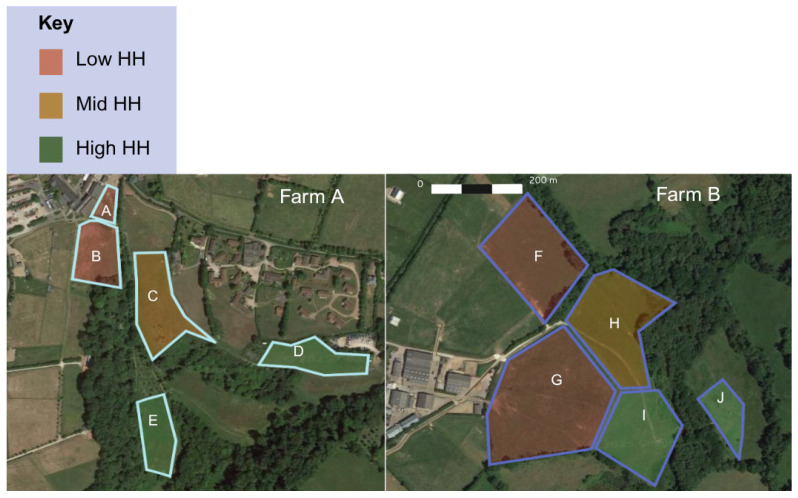
Categorisation of fields (A–J) based on culmination of Shannon’s diversity index, sward height, and structural variation using z-score classification. Image source: Google Earth © Google; imagery © Airbus, 18 June 2025.

Habitat heterogeneity varied considerably across fields, and Z-score HH classification grouped fields D, E, I, and J into high-HH, fields C and H into mid-HH, and fields A, B, F, and G into low-HH groups (Table 3; Figure 2).

### 3.3. Habitat Heterogeneity and Donkey Emotional State

There were two key dimensions of emotional state in the donkey herds, a mood axis (PC1) and an arousal or energy axis (PC2). PC1 (mood) accounted for 27.1% of the variance and was characterised by higher positive loadings for the descriptors happy (+0.15), at ease (+0.15), and relaxed (+0.11) and larger negative loadings for the descriptors agitated (−0.35), aggressive (−0.34), and uncomfortable (−0.34). PC2 explained 14% of the variance, with higher loadings reflecting more active and positive expressions such as relaxed (+0.47), happy (+0.45), at ease (+0.45), playful (+0.28), and friendly (+0.28) and lower loadings including withdrawn (−0.17), bored (−0.17), and apathetic (−0.12). Overall higher scores on mood and energy reflected positive emotional states of the herd. See Figure S5 and Table S3 in the Supplementary Materials for a full list of loadings on PC1 and PC2.

**Figure 3:**
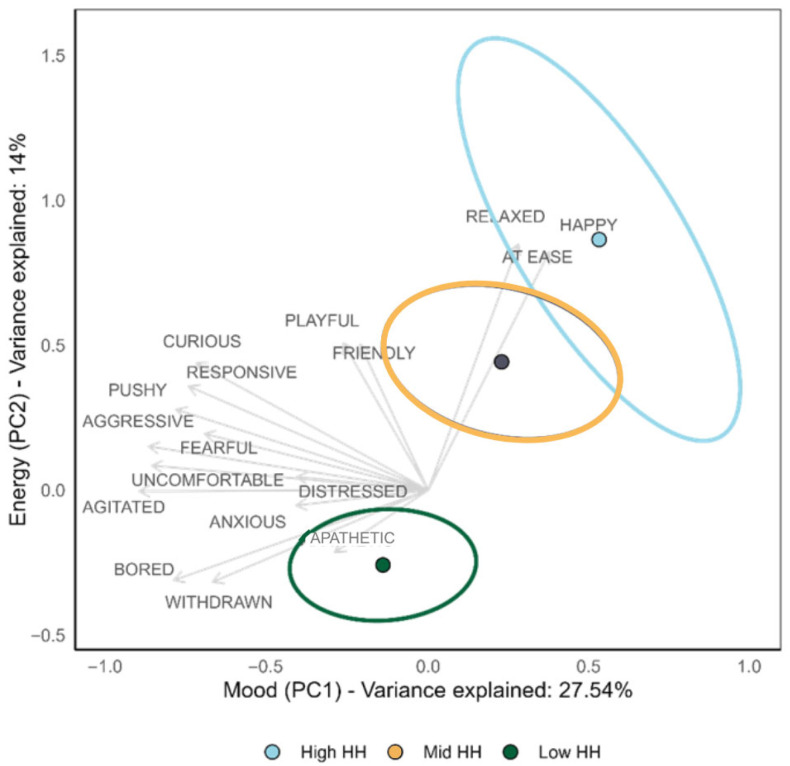
Biplot of PC1 (mood) and PC2 (energy) show clustering of emotional states by field habitat heterogeneity (HH). Points represent QBA scores with ellipses as confidence intervals. Vectors indicate emotional descriptors and their loadings within the energy and arousal principal component. Positive emotions happy, relaxed, and at ease are strongly loaded onto high habitat heterogeneity, while negative mood states are more aligned with low habitat heterogeneity.

Herd emotional state was clustered by field habitat heterogeneity (Figure 3). Donkeys in high-habitat-heterogeneity fields showed more relaxed mood and higher energy, whereas those in low-habitat-heterogeneity fields were associated with more negative mood states and lower energy. Mid-habitat-heterogeneity fields showed partial overlap with high-habitat-heterogeneity fields, suggesting some similarities but separation from low-habitat-heterogeneity fields.

#### Multivariate Testing for QBA Scores

Emotional expression differed significantly across all habitat heterogeneity categories (F_2,578_ = 5.57, R^2^ = 0.02, *p* < 0.001). Post hoc pairwise comparisons indicated that there were significant differences between low vs high habitat heterogeneity (p.adjusted = 0.01) and low vs mid habitat heterogeneity (p.adjusted = 0.003), while the high vs mid habitat heterogeneity comparison was not statistically significant (p.adjusted = 0.11).

Donkeys in high-heterogeneity fields were 14.2% more responsive (*p* = 0.001), 11.7% (*p* = 0.001) more friendly, and 13.5% (*p* = 0.033) more curious compared to those in low-habitat-heterogeneity fields and 15.8% (*p* = 0.006) more responsive and 12.9% (*p* = 0.011) more friendly than those in mid-habitat-heterogeneity fields. Donkeys in mid-habitat-heterogeneity fields were 7.3% (*p* = 0.011) more pushy than those in low-habitat-heterogeneity fields. See Table S4 in the Supplementary Materials for a full list of descriptor contributions to differences between habitat heterogeneity categories from permutation testing.

### 3.4. Components of Habitat Heterogeneity

Each component of habitat heterogeneity significantly contributed to overall donkey herd emotional expression. The average Shannon’s diversity index explained 0.84% of the variation (F = 5.03, *p* = 0.001), average sward height explained 1.17% (F = 6.96, *p* = 0.001), and structural variability explained 1.27% (F = 7.60, *p* = 0.001).

For mood (PC1), taller sward height (β = 0.0063, *p* < 0.01) was significantly associated with more positive mood states. Structural variation (β = 0.019, *p* = 0.24) and Shannon’s diversity index (β = −0.028, *p* = 0.56) were not a significant predictor of PC1.

For energy (PC2), higher structural variation (β = 0.562, *p* < 0.001) was strongly associated with increased energy levels. However, sward height (*p* = 0.05) and Shannon’s diversity index (*p* = 0.78) did not significantly predict PC2 scores.

**Figure 4:**
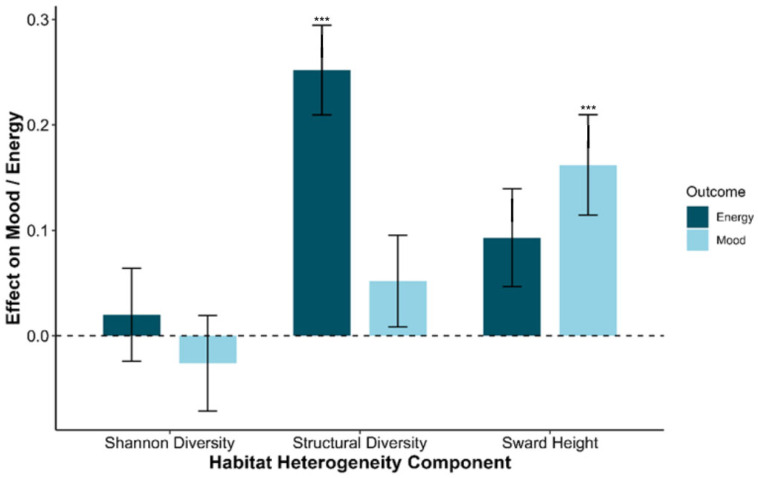
Standardised effect sizes of increasing habitat heterogeneity components on herd mood (PC1) and energy (PC2). Sward height had a positive effect on mood, while structural variation strongly predicted energy. Error bars represent 95% confidence intervals of the effect size. Asterisks denote significance levels.

In the scaled models, the greatest effect was structural variation on energy (β = 0.25, *p* < 0.001), while sward height showed a lower positive effect on mood (β = 0.16, *p* = 0.001) and an almost certain effect on energy (β = 0.09, *p* = 0.05) (Figure 4).

### 3.5. Effect of Farm and Herd

There were no differences in QBA scores between farm A and farm B (F_1,578_ = 2.01, R^2^ = 0.004, *p* = 0.07). Comparisons between herds indicated significant differences between herd 3 and herd 2 (F = 6.38, p.adjusted = 0.003) and between herd 2 and herd 1 (F = 4.13, p.adjusted = 0.003), while herd 3 and herd 1 did not differ (F = 2.07, p.adjusted = 0.195).

## 4. Discussion

No studies to date have explored how emotional expression as an indicator for donkey herd welfare is affected by environmental conditions, highlighting the novel approach taken here to understanding the variations in behaviour with changes in environment. In this study, we found that fields with higher habitat heterogeneity were associated with more positive energy and mood states in donkey herds. Specifically, donkeys were more responsive, curious, and friendly in heterogeneous environments. When considering the three different components of habitat heterogeneity separately, higher sward height was a main driver of positive mood, and higher structural variation was strongly associated with increased energy.

Complex environments may offer stimulation that encourages positive or interactive behaviours. These patterns are aligned with previous findings that greater vegetation variety, structure, and opportunities for interaction can support improved behaviours and physiological wellbeing [11,34]. Zoo enclosures may introduce different vegetation to encourage exploration and to allow animals to experience different habitats, promoting natural behaviours and reducing boredom [11]. Livestock have been observed moving and foraging more in fields with greater botanical diversity [34].

Structural variation was a key predictor of increased arousal and energy, suggesting that donkeys in more structurally diverse environments, such as those with hedges, scrub, and trees, exhibit more interactive and playful behaviours. While viewing video footage, assessors observed donkeys actively engaging with these features, such as reaching over a hedgerow and exploring field boundaries. Providing animals with a living space that has structures or features which they can choose to interact with can allow them to utilise more of their environment, which has behavioural and physiological benefits [11]. Shade from vegetation such as scrub and trees supports thermoregulation and provides resting opportunities [53], which may contribute to overall higher energy levels within the herd. Structural variation also increases opportunities for movement, for example, cattle move more when residing within a wooded habitat compared to grass paddocks [7]. Given that a lack of exercise is a common contributor in welfare concerns for donkeys in the UK, the use of naturally heterogeneous environments or those where features such as vegetation have been added to increase structural variation may assuage these concerns by increasing donkey movement and thereby improving their health. Donkeys evolved to travel and search for sparse foraging resources, and food-based enrichment which promotes movement can be an appropriate way to reduce the risk of health issues such as obesity and hyperlipemia [20].

We found that a higher sward height of grass was associated with calmer and more relaxed emotional states in donkeys. When forage is abundant, wild equids may spend more or less time feeding depending on the ecological context, indicating that forage quantity could influence behaviour [54]. Increased competition for resources and higher stress levels have been observed in other herbivores when resources are limited due to a high stocking density or reduction in sward height [14]. Therefore, it is possible that taller grass height leads to a more relaxed herd, as the perceived risk of limited food availability is lowered and intraspecific competition is reduced, particularly for larger-sized herds where the behavioural effects of limited resources may be amplified under higher stocking densities. Additionally, all fields studied had generally low browse levels, with only one offering unlimited browse, leading to a potential prioritisation of sward quantity by donkey herds.

Whilst we have focused on the impact that habitat heterogeneity has on donkey behaviour, it is likely that other environmental factors may have influenced donkey welfare. Given the nature of investigating donkey behaviour on active farms and our relatively small sample size, it was not possible to account for differences in field size, stocking density, or duration of time donkeys spent grazing on each field. Although each farm followed a predetermined grazing schedule, adjustments were made for practical needs and to accommodate poor weather conditions, which were not within the researchers’ control. Therefore, the amount of time spent in field may have differed between herds. In one of the fields, the fencing was adjusted to increase or decrease the field size based on the sward height. While this practice may have introduced a confounding effect related to sward height, field size alterations were minimal and not applied to any other fields in the study. The practice of moving donkeys when sward height dropped below 5 cm was applied consistently across all fields and grazing times, maintaining minimum forage availability. Best practice guidelines developed by The Donkey Sanctuary suggest a minimum grazing area of 0.5 acres per donkey, which is one of the factors used to develop grazing schedules. As a result, the total amount of grazing offered as part of their rotational grazing schedule over the season per herd met these requirements. Although stocking density was not formally accounted for in the current analysis, it is an important consideration and offers an interesting avenue for future research.

The amount of time that donkeys had access to fields differed between farms, with either 24 h access (farm A) or daytime access only (farm B), and equines can spend a high proportion of the night grazing if provided the option [38]. One farm had sanctuary visitors present, while the other had only Donkey Sanctuary staff. While the donkeys were habituated to visitors, their presence may have influenced donkey behaviour. These differing environmental aspects are acknowledged as potentially changing behaviour of donkey herds.

Post hoc analysis indicated some differences in underlying emotional profiles between herds. However, each herd was assessed independently, and no field was used by more than one herd on the same day. Farm did not significantly affect QBA scores, indicating that broader site-level differences (access times, visitor presence) were not a major source of variation in herd emotional expression. As the study examined how environmental diversity related to the emotional expression of herds within their respective fields, rather than comparing herds directly, the repeated measures of herd across multiple ecological measures minimise the influence of inherent herd differences.

The current study indicates there may be potential benefits to using a management approach which aims to support both animal welfare and biodiversity. Over-grazing (e.g., through over-stocking and/or insufficient rest periods) is likely to decrease sward height to unsustainable levels, and over time this will reduce plant and soil health, lower species diversity, and impair plant growth patterns [14,55]. Given that greater sward height was associated with calmer states in donkeys and high-habitat-heterogeneity fields were better for overall herd emotion, maintaining forage abundance and variability could support both welfare and ecosystem health. Pasture management guidelines, including those for donkeys, tend to suggest rotating herds before the sward is grazed below 5 cm, but the benefits of a taller swards have not previously been considered [56]. Increasing sward height must be balanced with other management practices and consider other factors such as grassland type and species composition to ensure all aspects of donkey welfare are being considered. Chapman et al. [57] found that grazers will select dietary species based on availability. Agriculturally improved pastures tend to be dominated by competitive species such as perennial ryegrass (*Lolium perenne*), which is higher in sugar content and carries a risk of contributing to obesity or laminitis in donkeys. By contrast, unimproved or semi-improved grassland with rough, tussocky sward of native, low-nutrient grasses may provide more suitable grazing for donkeys while also supporting healthier soils, diverse plant communities, and wider ecosystem services.

While botanical diversity was not a predictor of mood or energy, integrating sustainable pasture management practices that enhance grassland species and structural variation may support other aspects of donkey health. As donkeys are selective grazers [55,58], increased botanical diversity could encourage selective foraging, behavioural enrichment, nutritional benefits, and greater opportunity for movement. However, further research that quantifies donkey activity in relation to grassland composition and diversity is needed.

A structurally more diverse sward of native grass and forbs (i.e., broadleaf, non-woody herbaceous species) may also stimulate a wider range of foraging behaviours and expression of preferences, as donkeys pick and choose what to eat, potentially slowing their ingestion. By grazing on a variety of low-nutrient species, donkeys can potentially gain the benefits of grazing on a taller sward, such as reduced competition and a more fibrous diet, without the health risks that high-energy forage carries, such as obesity or laminitis. Currently, the available information about donkey palatability and preferences of herbaceous species is largely anecdotal. As a next step, we suggest preference and suitability studies to identify a suite of grassland species to include in pasture management plans, which can both promote donkey behaviour and welfare and support system health.

In a broad sense, heterogeneous environments, whether created or naturally occurring, may offer a wider range of ecological niches and, therefore, can support greater species diversity [35]. Donkeys are physiologically and behaviourally adapted to graze on nutrient-poor and widely distributed forage and show flexibility in their dietary choices between graze and browse species depending on the relative abundance of plant species [59]. In open habitats of conservation concern, such as coastal dunes and mountain pastures, this feeding behaviour makes donkeys effective conservation grazers when present at low densities and short grazing periods, as they can increase biomass and plant species diversity [60] and prevent the encroachment of scrub [42,59,61]. Lower stocking densities of grazers can increase habitat heterogeneity, indicating that applying sustainable grassland management principles can promote species diversity and increase structural variability, benefiting both the grazers and ecosystems [62]. With the study’s findings showing improved emotional expression in more heterogeneous environments, there is potential for a mutually beneficial relationship where donkeys are provided with a more stimulating environment, such as scrubby grassland, and, in turn, their browsing and grazing supporting broader ecosystem services including biodiversity.

Habitat heterogeneity is often underpinned by the complexity of the plant community, whereby the spatial and structural (particularly vertical) diversity contributes significantly to the biodiversity of the ecosystem as a whole [63]. As structural variation was found to increase energy in donkeys, the presence of browse and other physical features within in a donkey habitat is likely to have a positive effect on welfare as well as improving wildlife habitat and potentially ecosystem health. Most companion donkeys in the UK, however, are kept on species-poor grassland with little or no browse material available [25]. Donkey care guidelines should therefore include guidance on increasing their access to suitable browse through, for instance, planting of trees and hedgerows or allowing the natural regeneration of scrub species. Collaboration with ecology and conservation sectors would be beneficial here to ensure guidance is tailored to provide the most beneficial outcomes for donkeys and the environment.

## 5. Conclusions

This study provides evidence that habitat heterogeneity, particularly structural variation and sward height of donkey fields, has a positive effect on donkey welfare. These findings highlight the potential for environmentally enriched and structurally complex fields to enhance donkey welfare and should inform management practices for companion donkeys. Longitudinal studies or those in more controlled environments would further shed light on how different measures of field diversity impact behaviour.

## Figures and Tables

**Table 1:** Field features and management at time of study for farm A (fields A–E) and farm B (fields F–J).

Field	Donkeys Grazed	Browse Access	Area (m^2^)
**A**	End of July	No	1538
**B**	Early May	No	6604
**C**	End of August	Minimal	9943
**D**	Mid-May	Yes	6433
**E**	Early June	Yes	6240
**F**	Mid-June, Mid-July	Yes	17,908
**G**	Early June, Late July and August	Yes	31,426
**H**	Late May	Yes	17,570
**I**	Late May, Late June	Yes	14,462
**J**	Mid July	Yes	5406

**Table 2:** Demographics of herds used for study at time of study.

Herd	Farm	Herd Size	Age Range (Average)	Breed		Sex		Median Weight (kg)
				Standard	Miniature	Mare	Gelding	
1	A	53	3–21 (14.3)	49	4	34	19	187.00
2	B	90	5–25 (14.7)	86	4	46	44	193.05
3	B	72	6–24 (14.9)	72	0	34	38	196.71

**Table 3:** Mean (95% CI) values of structural variation (indexed 1–4), sward height (cm), and Shannon’s diversity index for fields categorised into low-, mid-, and high-habitat-heterogeneity (HH) groups based on Z-scores.

	Low HH	Mid HH	High HH
Structural variation	1.80 (1.76–1.84)	2.74 (2.64–2.84)	3.16 (3.09–3.23)
Sward height (cm)	12.25 (11.69–12.81)	7.40 (6.98–7.82)	19.98 (18.99–20.97)
Shannon’s diversity	1.10 (1.08–1.12)	1.27 (1.22–1.32)	1.24 (1.19–1.29)

## Data Availability

Data is available upon request to the corresponding author.

## Supplementary Materials

The following supporting information can be downloaded at: https://www.mdpi.com/article/10.3390/ani15233421/s1, Table S1: Criteria for assessing structural variation in fields based on the availability of browse, trees, and field features; Figures S1–S4: Visual representation of structural variation scores 1–4, indicating differences in browse, tree, and hill feature access; Table S2: List of QBA descriptors and definitions based on Minero et al. 2016 [41]; Figure S5: Amount of variance captured in each PC; Table S3: Loadings of QBA descriptors on PC1 and PC2; Table S4: Results of a SIMPER analysis to identify percentage contribution of emotional descriptors to differences in overall emotional state of donkeys in fields of high, mid, and low habitat heterogeneity.
