# Supplementary material for: Pasture Heterogeneity Improves Donkey Welfare: Effects of Structural Variation, Species Diversity, and Sward Height on Herd Emotional States
Source: Animals (Basel). 2025 Nov 27;15(23):3421. doi: 10.3390/ani15233421 (PMC12691164; doi:10.3390/ani15233421)
Supplement: Supplementary file 1 [file animals-15-03421-s001.zip › animals-3985322-supplementary.pdf]

Supplementary Materials

Table S1. Criteria for assessing structural variation of field based on the availability of browse, trees, and field features

**1**	Flat grassland no browse or tree access
**2**	Limited browse (can be consumed by herd within a short period of time) and/or tree access within field or just along perimeter, some small hilly features
**3**	Browse and/or tree access along perimeter or within field, more hill features such as a larger hill or larger part of field containing hill
**4**	Unlimited browse and/or tree access along perimeter or within field, distinct hilly features within entirety of field

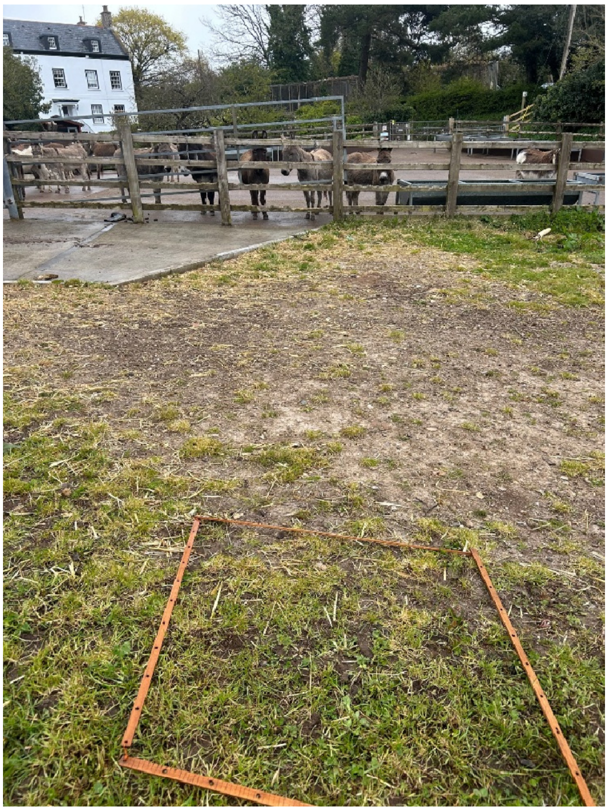

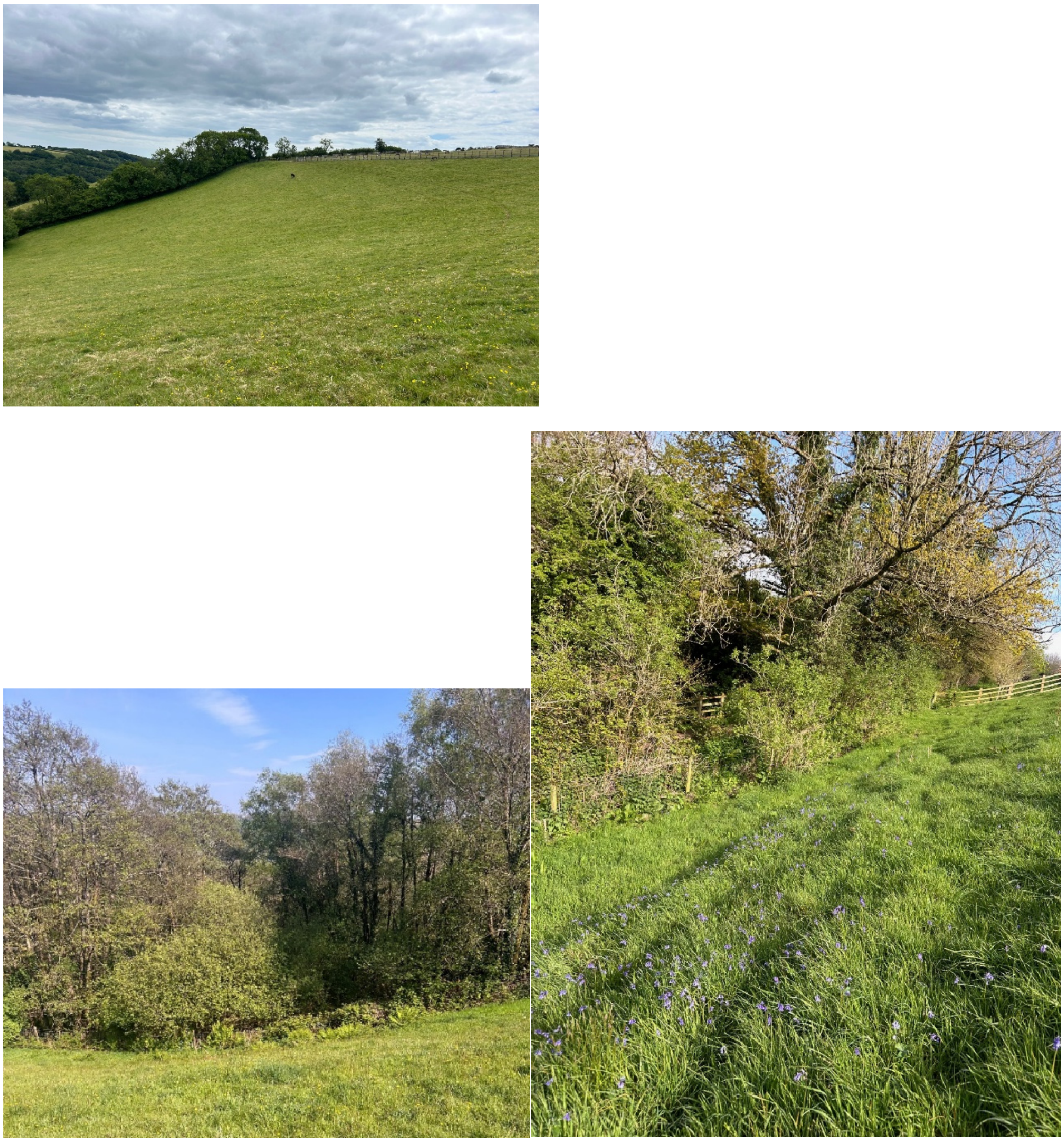

Figures S1–S4. Visual representation of structural variation scores 1–4, indicating differences in browse, tree, and hill feature access.

Table S2. List of QBA descriptors and definitions based on Minero et al. 2016.

Descriptor	Definition
At ease	Calm with other animals, interested but with appropriate calm reactions to changes, carefree, resting in close proximity to other animals. Maintains normal relaxed behaviour around people or other animals.
Curious	Inquisitive, interested in surroundings, interacts with new or novel objects, people or animals, investigative, explores environment objects or people.
Friendly	Companionable, affectionate, sociable; not hostile, positive reactions towards another person or animal, seeks attention from human (ie the donkey approaches another animal/ person and expresses grooming behaviour, may sniff or interact in some way).
Happy	Content, feeling, showing or expressing joy, pleased, lively, playing, satisfied, relaxed alertness to environment, interested in surroundings.
Playful	Very active on own or with others, actively seeking play opportunities, frisky/frolicsome, playing with toys/objects, reciprocal play fighting; trying to encourage other animals in play.
Relaxed	Not tense or rigid, easy-going, calm, tranquil, standing still while resting a foot, normal level head position, minimal relaxed tail swishing, calm awareness/interest in surroundings.
Responsive	Reacts appropriately to behaviour of other animals and people, active, acknowledging, receptive, aware of the environment, shows appropriate responses to stimuli, vocalising.
Apathetic	Having or showing little or no emotion, uninterested, indifferent, not responsive to stimuli, little or no ear movement, ears low, little or no interaction with other donkeys, depressed, not moving or moving slowly, low head position, appearing to behave stoically, shutdown.
Agitated	A negative emotional state, highly active, restless, fidgety, excited, worried/upset, disturbed, in a bad mood, annoyed, stereotypy (weaving, fence pacing, irritated by flies, head shaking, muscle twitch, rapid rigid tail swishing, persistent ear movements.
Aggressive	Hostile, attacking, defensive, disruptive, angry, wants to fight/attack another donkey, intention

	to harm, displays kick threats, bite threats, head tossing, ears flat-back against neck, vigorous tail swishing towards other animals or people, physical contact, biting, chasing, kicking.
Anxious	Worried/tense, troubled, apprehensive, distressed, jumpy, nervous, watchful, responsive to a possible threat/danger (ie looking around/vigilant, moving ears, opposite of at ease).
Distressed	Upset, afflicted, distraught, worried, defensive behaviours, high levels of movement and resistance to restraint (ie high resistance to handling, attempts to escape, abnormal defecation, rearing up).
Fearful	Startled, afraid, hesitant, timid, uneasy, may often be linked to something going on in the environment, flight response, move or run away, back up, refuse to move further, may show defensive behaviour, bite threats, kick threats or avoidance behaviour such as pushing handler.
Pushy	Assertive or forceful, gaining access to physical resource such as food/water/human contact by physical means, displacement of another donkey, head butt out the way.
Uncomfortable	Painful, irritated, repetitive behaviour such as rubbing, biting or itching particular spots of the body, (eg shooing away flies, trying to remove a too tight head collar) foot stamping, weight shifting, showing conflict or uncertainty with tense muzzle/ back or tucked tail.
Withdrawn	Unsociable, introverted, reclusive, not wanting to communicate with other animals or people, uncommunicative, reserved, unresponsive, not searching for contact with others, solitary.
Bored	Wandering aimlessly without direction or intention, but not relaxed or resting. Behaviour such as wood chewing, unnatural repetitive behaviour, general lethargy not caused by excessive workload

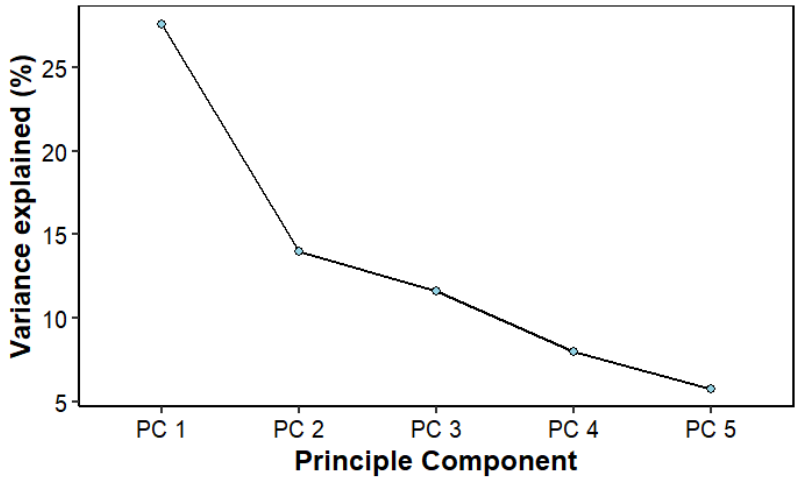

Figure S5. Amount of variance captured in each PC

Table S3. Loadings of QBA descriptors on PC1 and PC2

Descriptor	PC1 Mood	PC2 Energy
Happy	0.148	0.453
At Ease	0.148	0.451
Relaxed	0.110	0.468
Friendly	-0.083	0.277
Playful	-0.104	0.280
Apathetic	-0.114	-0.118
Distressed	-0.161	0.025
Anxious	-0.162	-0.029

Descriptor	PC1 Mood	PC2 Energy
Withdrawn	-0.264	-0.175
Fearful	-0.274	0.107
Curious	-0.284	0.242
Responsive	-0.294	0.198
Pushy	-0.309	0.153
Bored	-0.312	-0.171
Uncomfortable	-0.338	0.047
Aggressive	-0.343	0.083
Agitated	-0.354	-0.002

Table S4. Results of a SIMPER analysis to identify percent contribution of emotional descriptors to differences in overall emotional state of donkeys in fields of high, mid, and low habitat heterogeneity. Asterisks indicate significance levels: *p < 0.05; **p < 0.01; ***p < 0.001.*

Comparison	Emotional Descriptor	Mean Contribution (%)	p-value	Direction
High vs Mid HH	Responsive	82.7	0.006**	High HH
Hih vs Mid HH	Curious	77.2	0.205	High HH
High vs Mid HH	Friendly	67.8	0.011**	High HH
High vs Mid HH	Uncomfortable	50	0.766	High HH
High vs Mid HH	Relaxed	46.1	0.946	Mid HH
High vs Mid HH	Happy	43.3	1	High HH
High vs Mid HH	Agitated	36.4	0.907	Mid HH
High vs Mid HH	Playful	24.6	0.056	High HH
High vs Mid HH	Pushy	23.7	0.93	MidHH
High vs Mid HH	At.Ease	23	0.999	HighHH
High vs Mid HH	Aggressive	20	0.959	MidHH
High vs Mid HH	Fearful	9.1	0.966	MidHH
High vs Mid HH	Anxious	8.8	0.817	MidHH
High vs Mid HH	Bored	6.4	1	MidHH
High vs Mid HH	Withdrawn	2.7	1	MidHH
High vs Mid HH	Distressed	1.3	0.816	MidHH
High vs Mid HH	Apathetic	0.3	0.882	MidHH
Low vs High HH	Responsive	84.2	0.001*	HighHH
Low vs High HH	Curious	80	0.033*	HighHH
Low vs High HH	Friendly	69.5	0.001***	HighHH
Low vs High HH	Happy	57.6	0.6	HighHH
Low vs High HH	Uncomfortable	52.9	0.566	LowHH
Low vs High HH	Relaxed	51.7	0.521	HighHH
Low vs High HH	Agitated	37.6	0.856	LowHH
Low vs High HH	Bored	30.4	0.884	LowHH
Low vs High HH	At Ease	26.1	0.974	HighHH
Low vs High HH	Withdrawn	22	0.82	LowHH
Low vs High HH	Playful	21.4	0.139	HighHH

Low vs High HH	Aggressive	19.6	0.99	LowHH
Low vs High HH	Pushy	18	1	LowHH
Low vs High HH	Fearful	9.8	0.961	LowHH
Low vs High HH	Anxious	9.1	0.768	LowHH
Low vs High HH	Distressed	1.9	0.551	LowHH
Low vs High HH	Apathetic	0.9	0.565	LowHH
Low vs Mid HH	Curious	72.9	0.945	MidHH
Low vs Mid HH	Responsive	72	0.999	MidHH
Low vs Mid HH	Happy	59.6	0.189	MidHH
Low vs Mid HH	Friendly	59.5	0.969	MidHH
Low vs Mid HH	Uncomfortable	52.8	0.79	LowHH
Low vs Mid HH	Relaxed	50.4	0.944	MidHH
Low vs Mid HH	Agitated	42	0.468	LowHH
Low vs Mid HH	Pushy	34.6	0.011**	MidHH
Low vs Mid HH	Bored	33	0.998	LowHH
Low vs Mid HH	At Ease	30.8	0.498	MidHH
Low vs Mid HH	Aggressive	30.1	0.154	MidHH
Low vs Mid HH	Withdrawn	23	0.999	LowHH
Low vs Mid HH	Playful	17.5	0.2	MidHH
Low vs Mid HH	Fearful	16.2	0.298	LowHH
Low vs Mid HH	Anxious	11.2	0.399	LowHH
Low vs Mid HH	Distressed	2.6	0.593	LowHH
Low vs Mid HH	Apathetic	1.2	0.69	LowHH
